# Anthropometric and Dietary Factors as Predictors of DNA Damage in Obese Women

**DOI:** 10.3390/nu10050578

**Published:** 2018-05-08

**Authors:** Marta Włodarczyk, Beata Jabłonowska-Lietz, Wioletta Olejarz, Grażyna Nowicka

**Affiliations:** 1 Department of Biochemistry and Pharmacogenomics, Faculty of Pharmacy with Division of Laboratory Medicine, Medical University of Warsaw, Banacha 1B, 02-097 Warsaw, Poland; wolejarz@wum.edu.pl (W.O.); gnowicka@wum.edu.pl (G.N.); 2 Centre for Preclinical Research, Medical University of Warsaw, Banacha 1B, 02-097 Warsaw, Poland; 3 Centre of Promotion of Heathy Nutrition and Physical Activity, National Food and Nutrition Institute, Powsinska 61/63, 02-903 Warsaw, Poland; bjablonowskalietz@gmail.com

**Keywords:** DNA damage, obesity, dietary intake, vitamin C, vitamin E

## Abstract

Enhanced DNA damage and disturbances in DNA repair mechanisms are reported to be involved in the pathogenesis of chronic diseases like obesity, atherosclerosis, metabolic syndrome, diabetes, and cancer. The aim of the present study was to evaluate whether anthropometric factors and dietary habits are related to endogenous DNA damage. One hundred and fourteen premenopausal, apparently healthy women were included in the study: 88 obese individuals and 26 controls. The comet assay was used to measure basal DNA damage. Biochemical measurements included lipids, apolipoproteinAI, fasting insulin, glucose, and C-reactive protein high sensitivity (CRP-hs). Dietary intakes were assessed by 3-day food records. The mean level of DNA damage was almost two times higher in obese than in non-obese women (*p* < 0.001). Regression modeling showed that body mass index (BMI), daily intakes of energy, and vitamin C are key predictors of variance in basal DNA damage. Our data demonstrate the impact of obesity-associated inflammation on DNA damage and indicate that regardless of obesity, the level of DNA damage can be reduced by adequate intakes of vitamins C and E. It suggests that particular attention should be paid to the content of antioxidants in the diet of obese people and further studies are needed to modify dietary guidelines to prevent DNA damage in obese individuals.

## 1. Introduction

DNA contains information needed for cells to function. However, DNA is exposed to damage by metabolic byproducts and environmental factors like pollutions and ultraviolet light [1,2,3]. As long as many of these damages can be undone by repair mechanisms, cells and organs can function properly [4]. Otherwise DNA damage can lead to oxidative stress, inflammation, genotoxicity, mutations, and disturbances in cell metabolism [5,6]. Accumulation of DNA damage was found to be linked to aging and the onset of age-related disorders including cardiovascular disease, diabetes, and malignant transformation [7].

Obesity has been recognized as an important health problem that increases the risk for chronic diseases like atherosclerosis, metabolic syndrome, diabetes, and cancer [8,9]. Excessive accumulation of triglycerides in adipocytes promotes inflammation in adipose tissue. The inflammatory response in visceral adipose tissue results in the development of insulin resistance and disturbances in glucose and lipid metabolism. DNA damage in obese individuals may enhance obesity-associated inflammatory processes as well as affect cellular metabolism, resulting in disturbances in systemic metabolic homeostasis and the endocrine system, and promote the development of metabolic disorders and obesity-associated comorbidities [10].

It was shown that weight loss through dietary changes reduced oxidative stress and markers of inflammation such as C-reactive protein (CRP), tumor necrosis factor-α (TNF-α), and interleukin 6 (IL-6) [11]. In addition to endogenous antioxidant systems, a diet rich in antioxidants can protect DNA and increase cell resistance against oxidative stress [12,13]. Diets rich in fruits and vegetables were found to lower the risks of metabolic diseases and cancer [14,15]. Moreover, in the maintenance of DNA stability, both the amount and quality of dietary fat seem to be important. A PUFA (polyunsaturated fatty acid)-rich diet was found to be associated with reduced DNA damage [16]. On the other hand, some studies suggest that PUFAs can increase oxidative DNA damage through lipid peroxidation [17,18] while SFAs (saturated fatty acids) may promote cell transformation by negatively regulating the DNA damage response pathway and contribute to tumor progression [19].

The aim of the present study was to assess the role of anthropometric and dietary factors as determinants of DNA damage in obese and non-obese women.

## 2. Materials and Methods

### 2.1. Study Participants

The study group consisted of 114 apparently healthy, premenopausal women. All subjects were Polish Caucasians from the Warsaw region. The patients were consecutively recruited between January 2010 and January 2013 on the basis of clinical assessments from subjects who had been directed to the Outpatient Clinic at the National Food and Nutrition Institute in Warsaw due to obesity treatment or a routine general health screening. The recruited subjects were non-smoking (for at least 5 years), had no signs or symptoms of renal and hepatic disorders, endocrine disorders (e.g., disease of the thyroid, parathyroid, Cushing’s syndrome, polycystic ovary syndrome), autoimmune diseases, cancer, and no history of alcoholism. Exclusion factors were also body mass index (BMI) > 39 kg/m^2^, menopause, pregnancy, or lactation.

Women within the last 3 months before the study were not receiving medications known to influence plasma lipid and glucose levels and did not use hormonal therapy as well as did not report the chronic use of dietary supplements and anti-inflammatory drugs. The study was conducted according to the guidelines laid down in the Declaration of Helsinki and all procedures involving human subjects were approved by the Local Ethics Committee. Written informed consent was obtained from all of the registered volunteers.

### 2.2. Anthropometric Measurements

All subjects underwent a comprehensive medical evaluation including medical history, physical examination, and measurement of anthropometric parameters: body weight, body height, waist circumference, and hip circumference according to standardized procedures routinely performed in the Outpatient Clinic at the National Food and Nutrition Institute. The body waist circumference was measured at the midpoint between the lower margin of the last rib cage and the top iliac crest by using a flexible inch tape. An analysis of the body composition by bioelectrical impedance was performed using TANITA MC180MA (Tanita Corporation, Tokyo, Japan) and according to built-in algorithms the following parameters were obtained: body fat mass (kg), free fat mass (kg), and fat mass (%). The free fat mass referred to all of body components (including total body water, muscle mass, bone mass) except fat mass. Measurements were taken in the morning, after an overnight fasting, at the same day or the day before blood samplings. GMON Tanita Professional software (version 3, Tanita Corporation, Tokyo, Japan) was used for the analysis. Based on anthropometric measurements the BMI and waist-hip-ratio (WHR) indexes were calculated [20]. Obesity was classified according to World Health Organization criteria [21] i.e., subjects with BMI ≥ 30 kg/m^2^ were considered obese.

### 2.3. Dietary Intake Assessment

Dietary intake was assessed by two 3-day food records (2 non-sequential week days and 1 weekend day). Subjects had previously been trained by a professional dietician on how to report food intake and assess the consumed sizes of foodstuff dishes and meal portions based on a photographic album of food products and dishes. Recorded data were analyzed using the Diet 5 software (National Food and Nutrition Institute, Warsaw, Poland, 2011) based on Polish Foodstuff Composition and Nutritional Value Tables [22,23]. In order to assess the dietary intake of macronutrients, their proportional (%) share of supplied calories was calculated.

### 2.4. Blood Analyses

Blood was collected after night fasting from all subjects in commercially available vacuum tubes and analyzed on the same day. Serum concentrations of total cholesterol, HDL-cholesterol (high density lipoprotein cholesterol), triglycerides, glucose, and insulin were measured using standard techniques in a certified laboratory for clinical chemistry at The National Food and Nutrition Institute. LDL-cholesterol (low density lipoprotein cholesterol) was calculated according to the Friedewald formula [24]. The serum levels of apolipoprotein AI (apo AI) were measured using monoclonal antibodies against apo AI (Pointe Scientific, Canton, MI, USA) by the immunoturbidimetric method. Serum high sensitivity C-reactive protein (CRP-hs) concentration was measured using a commercially available enzyme-linked immunosorbent assay (ELISA) (Immundiagnostik AG, Bensheim, Germany). Homeostasis Model Assessment for Insulin Resistance, HOMA-IR index, a commonly used marker of insulin resistance, was calculated using the formula: HOMA-IR = fasting glucose levels (mmol/L) × fasting insulin levels (μU/mL)/22.5 [25].

### 2.5. Comet Assay

DNA integrity was evaluated by the alkaline single-cell gel electrophoresis (comet assay), in accordance with Singh et al. [26], with some modifications [27]. Lymphocytes were freshly isolated from 1 mL heparinized blood by centrifugation in a density gradient; then 50 μL of lymphocytes (1–3 × 10^5^ cells/mL) was distributed with 50 mL of 2% low-melting-point agarose on a microscope slide precoated with 0.5% normal agarose. The slides were immersed for 1 h in a freshly prepared cold (4 °C) lysis solution (2.5 M NaCl, 100 mM Na2EDTA, 10 mM Tris, pH 10.0–10.5) with 1% Triton X-100 added just before solution use. After lysis, the slides were placed in a horizontal gel electrophoresis tank with fresh alkaline electrophoresis buffer (300 mM NaOH, 1 mM Na2EDTA, pH > 13.0) and left in the solution for 40 min at 4 °C. Electrophoresis was conducted at 4 °C for 20 min at 35 V (1 V/cm) and 300 mA. Subsequently, slides were washed three times with a neutralizing solution (0.4 M Tris, pH 7.5), stained with DAPI (4′,6-diamidino-2-phenylindole, 20 μg/mL), and analyzed with a Nikon Eclipse 50i fluorescence microscope at 400× magnification. On each slide, 100 comets were scored using Lucia Comet Assay software version 4.81 (Laboratory Imaging, Prague, Czech Republic). All experiments were conducted in duplicate using three blood samples taken from each subject. In each electrophoresis, two duplicate samples from three donors were run simultaneously. Of the data obtained, % DNA in the tail was chosen for further analysis as a DNA damage parameter. The chemicals were supplied by Sigma-Aldrich.

### 2.6. Statistical Analysis

Data were analyzed with the Statistica 12.0 program (Statsoft, Krakow, Poland). Variables were compared with the normal distribution using the Shapiro-Wilk test. Differences between groups were compared using the Mann-Whitney *U*-test. Univariate analyses were performed by Spearman’s nonparametric correlation test. The statistical significance threshold was set at *p* > 0.05. The effects of demographic characteristics (age, BMI) and dietary factors (intakes of energy, SFA as % energy, and selected vitamins) on DNA damage were calculated using hierarchical multiple regression analyses.

## 3. Results

Baseline characteristics of subjects enrolled in the study are shown in Table 1. Subjects complying with all aspects of the study design included 114 participants: 88 obese (BMI ≥ 30 kg/m^2^) and 26 non-obese women (BMI < 30 kg/m^2^), aged 39 ± 6 years (24–52 years). Among non-obese participants (controls), the prevalence of overweight (defined as BMI 25–29.9 kg/m^2^) was 65%, i.e., 20 subjects had BMI in the range of 25.30–28.60 kg/m^2^ (mean 27.92 ± 1.58 kg/m^2^) and 6 subjects had BMI < 25 kg/m^2^ (mean 21.00 ± 1.14 kg/m^2^, range of 19.11–22.20 kg/m^2^) Normal weight and overweight women were of similar age (mean 39 ± 10 and 40 ± 6 years, respectively). Similar levels of CRP-hs were observed among normal weight and overweight women: 1.42 ± 0.64 and 1.61 ± 1.05 mg/L, respectively. In overweight women, higher levels of DNA damage (% DNA in tail) were observed in comparison to normal weight women (2.55 ± 0.59 vs. 1.74 ± 0.34, respectively; *p* = 0.0003). However, due to the small size of the groups, overweight and normal weight participants were subjected to statistical analyses together. In obese women, significantly higher serum concentrations of total and LDL cholesterol, and glucose were recognized (Table 1). The mean basal level of DNA damage in the studied group was 4.50 ± 2.47 (% DNA in tail), however, the mean level of DNA damage was almost two times higher in obese than in non-obese women (*p* < 0.0001) and was associated with significantly enhanced serum CRP-hs concentrations. A positive correlation between serum CRP-hs (mg/L) and DNA damage in studied women was recognized (*R* = 0.457, *p* < 0.05).

As presented in Table 2, obese women were characterized by higher total energy (kcal), total fat (% energy), and saturated fat (% energy) intake. Consumption of carbohydrates (% energy), PUFA (% energy), and vitamin E (mg) was lower in studied obese women than in controls.

Spearman correlation was used to identify relationships between basal DNA damage (% DNA in tail), anthropometric and biochemical parameters, and dietary factors (Table 3). In the present study, no relationship was noted between DNA damage and age in the obese group, however, such an association was observed in controls. DNA damage was significantly associated with body weight, BMI, and fat mass, indicating an adverse effect of the enhanced accumulation of adipose tissue on DNA integrity. This is supported by an observed association between WHR and DNA damage in the control women. Significant positive correlations between DNA damage and total energy intake as well as percent of energy from total fat and saturated fat were recognized. Intake of vitamins C and E was inversely related to DNA damage, indicating the impact of these antioxidants on maintaining DNA integrity in the studied subjects. Furthermore, a positive correlation between DNA damage and dietary SFA intake was observed in all subjects and among obese women (*p* < 0.05). A positive association between DNA damage and serum CRP-hs concentration was found and a stronger correlation was observed among obese women than in the controls, suggesting an impact of obesity-associated inflammation. Total serum cholesterol, LDL-cholesterol, triglycerides, and glucose concentrations were also associated with DNA damage.

Hierarchical linear regression analysis was conducted to investigate the impact of anthropometric and dietary variables on DNA damage (criterion variable). Age and BMI were entered in the first step (Table 4, model 1) and a significant impact of BMI on DNA damage was recognized. When total energy intake (kcal/day) was entered in the second step, the association between BMI and DNA damage was retained and this model explained 30.3% of the variance in DNA damage. Model 3 showed the lack of significant association between SFA (% energy) and DNA damage, therefore SFA was not included in further analyses.

In model 4, vitamin E was entered and in model 5 vitamin C was entered, and in both models, the relationships between BMI and total energy and DNA damage were retained. Vitamin C and E were negatively associated with DNA damage and these models explained 34.5% and 32.6% of the variance in DNA damage, respectively. Inclusion of β-carotene and retinol did not improve our analyses and did not explain a significant amount of variance in DNA damage regardless of studied variables (data not shown). The final model 6 included age, BMI, total energy, and vitamin C and vitamin E intakes. In this model, vitamin E was no longer significantly related to DNA damage. The analyses revealed that BMI, energy intake, and vitamin C intake are key factors affecting the level of DNA damage.

## 4. Discussion

The comet assay is one of the methods used to evaluate DNA damage in individual cells and is a widely used tool for monitoring genome stability and assessing the role of oxidative stress in human diseases, aging processes, and mutagenesis, [3,28] as well as to investigate DNA damage in different cell types in response to a range of agents including drugs and foods [29]. The alkaline version of the comet assay detects single-strand DNA breaks, alkali-labile sites, crosslinks, and incomplete DNA repair sites in individual cells. The data reviewed by Valverde and Rojas [30] reflect the increasing role of the comet assay in human biomonitoring studies in providing useful information about genome stability. Peripheral blood lymphocytes can be easily obtained, and the DNA damage detected in these cells has been recognized to mimic the DNA damage in other cells [3,31]. The level of carcinogen-DNA adducts in blood lymphocytes has been shown to correlate with tobacco carcinogen-induced damage in human lung tissues [32]. Moreover, leukocytes are also recognized as surrogate cells, which are used for providing information about oxidative stress (measured as the level of 8-oxoGua) in other tissues [33]. DNA damage has been widely discussed in carcinogenesis. [34]. Levels of DNA damage have been recognized as a prognostic marker identifying patients with poor response to chemotherapy [35]. Moreover, the association between DNA damage and atherosclerosis has been proposed. The accumulation of DNA damage appears in human atherosclerotic lesions [36,37]. The reduction of DNA damage has been suggested to be crucial for the prevention of atherosclerosis and related cardiovascular diseases [38].

Cellular DNA damage has been recognized to be related to aging [39,40]. In the present study, a significant association between DNA damage in lymphocytes and age was observed in control women but not in similar aged obese women, indicating the impact of obesity-associated factors that attenuate the effect of age. The massive accumulation of triglycerides in adipocytes leads to adipocyte hypertrophy inducing mitochondrial dysfunction, enhanced production of reactive oxygen species (ROS), proinflammatory cytokines and the development of chronic inflammation, and disturbances in insulin action and glucose metabolism [41]. Inflammation and metabolic disturbances can cause DNA damage. Previously, accumulation of DNA damage has been reported in patients with cardiovascular diseases, diabetes mellitus, obesity and cancer [42,43,44]. In the present study, DNA damage levels in obese women were more than two times as high in comparison to control women and a significant correlation between DNA damage and serum CRP-hs concentration was recognized. This supports the hypothesis regarding the effect of obesity and obesity-associated inflammation on DNA damage. On the other hand, cellular response to DNA damage can result in enhanced production of inflammatory cytokines and disturbances in cell metabolism. It was reported that responses to DNA damage include irreversible cell-cycle arrest [45], activation of nuclear factor kappa B (NF-kB) [46,47,48,49], and overexpression of p53 [50], which can induce adipocyte differentiation and hypertrophy, can cause inflammation, and can impair glucose metabolism and promote the development of obesity, systemic insulin resistance, and diabetes [51,52].

There is no doubt that level of energy intake and dietary pattern influence the development of obesity and obesity-associated metabolic disturbances [53] and can also affect genome stability [54]. The total daily energy intakes in our groups were in the range reported in other studies [55,56]. We observed that daily intakes of energy, SFA, and vitamins C, E, β-carotene, and retinol affect levels of DNA damage. However, multiple linear regression analyses showed that after adjustment for age, BMI, and total energy intake only the content of vitamins C and E in the diet was associated with DNA damage. It has been shown that diets rich in fruits and vegetables protect the body cells from oxidative damage [57] while low intake of antioxidants increases oxidation of biomolecules [58,59]. The Mediterranean diet, rich in antioxidants, including vitamins C and E, has been shown to reduce oxidative damage to lipids and DNA [60]. In in vitro experiments, α-tocopherol and ascorbic acid protected isolated cardiomyocytes against oxidative damage [61]. Additionally, in healthy subjects, vitamin C and α-tocopherol negatively correlated with 8-oxodG and 8-oxoGua, which are accepted markers of oxidative DNA damage [62]. Therefore, the present study indicates that more attention should be paid to the content of major antioxidant vitamins and probably also to other antioxidants in the diet of obese people in order to prevent DNA damage, which can enhance the risk of obesity-related comorbidities.

Previous studies indicated the role of total fat as well as saturated and unsaturated fatty acids intake in the prevention of obesity-associated diseases [63,64,65] and their effects on DNA damage [66]. Assessment of dietary intake of macronutrients in our study showed that diet of obese patients contained more energy from fat and SFA and less energy from PUFA compared to the non-obese control group. Moreover, the amount of SFA consumed appeared to be an important determinant of basal DNA damage. However, this effect of dietary SFA on DNA damage disappeared after adjustment for total energy intake. These findings are in line with recommendations to reduce the consumption of total energy as well as SFA and to deliver with diet adequate amounts of MUFA and PUFA, especially n-3 PUFA, whose role in preventing the inflammatory process is well accepted [66,67].

Our study does have some limitations. The first limitation is a small sample size. We studied only women aged 24–52 years. Therefore, data for men as well as younger and older populations containing subjects of both genders and with wide ranges of BMI values are needed. Furthermore, dietary intakes of nutrients of interest should be confirmed by the analyses of specific intake and/or status biomarkers. Other biomarkers, such as 8-oxodG (8-oxo-2′-deoxyguanosine) and 8-oxoGua (8-oxoguanine), which are accepted markers of oxidative DNA damage and isoprostanes, as markers of oxidative stress, should also be assessed to give a deeper insight into the effect of dietary antioxidants and their impact on DNA damage prevention. Assessment of markers of DNA repair pathways should also be performed in further research. Furthermore, in obese patients, reduced energy intake and associated body weight loss was reported to reduce DNA damage [68]. However, a recently published systemic review showed the inconsistent effects of weight loss on telomere length and DNA repair [69]. Therefore, further studies are needed to establish the effect of weight loss on DNA damage and DNA repair.

In summary, our data demonstrate the impact of adiposity and obesity-associated inflammation and support the effect of dietary factors promoting obesity on DNA damage, and for the first time indicate that regardless of obesity, the level of DNA damage can be reduced by adequate intakes of antioxidant vitamins, especially vitamin C. The results of our study suggest that particular attention should be paid to the content of antioxidants in the diet of obese people. Given the high incidence of obesity and obesity-associated diseases, further studies are needed to determine the extent to which dietary guidelines for vitamin C and other antioxidants intake should be modified to prevent DNA damage in obese individuals.

## Figures and Tables

**Table 1:** Characteristics of the study participants.

Variable	Controls (*n* = 26)	Obese (*n* = 88)
Age (years)	39.96 ± 7.07	38.15 ± 5.55
Height (cm)	164.58 ± 7.35	163.96 ± 6.22
Body weight (kg)	72.25 ± 13.83	87.98 ± 9.66 ***
BMI (kg/m^2^)	26.33 ± 3.32	33.05 ± 2.17 ***
WHR	0.837 ± 0.07	0.875 ± 0.06 *
Systolic blood pressure (mmHg)	127.71 ± 19.00	125.56 ± 17.22
Diastolic blood pressure (mmHg)	82.29 ± 9.67	82.19 ± 8.19
Fat mass (%)	32.92 ± 5.38	37.78 ± 3.21 ***
Fat mass (kg)	25.12 ± 8.24	34.59 ± 6.62 ***
Free fat mass (kg)	47.13 ± 9.75	52.22 ± 4.96 ***
Total Cholesterol (mg/dL)	190.42 ± 30.97	209.83 ± 36.52 *
HDL cholesterol (mg/dL)	60.23 ± 10.34	58.86 ± 15.26
LDL cholesterol (mg/dL)	110.85 ± 29.14	128.17 ± 32.92 *
Triglycerides (mg/dL)	93.93 ± 29.14	113.23 ± 48.40
Total Cholesterol/HDL cholesterol	3.14 ± 0.66	3.88 ± 1.30 *
Triglycerides/HDL cholesterol	2.07 ± 1.35	2.13 ± 1.20
Glucose (mg/dL)	84.20 ± 8.58	88.12 ± 8.49 *
Insulin (mU/mL)	8.91 ± 2.59	11.63 ± 5.20
HOMA	1.92 ± 0.60	2.53 ± 1.19
Apolipoprotein AI (mg/dL)	163.30 ± 18.34	159.55 ± 30.09
CRP-hs (mg/L)	1.57 ± 0.96	3.94 ± 3.51 ***
DNA damage (%)	2.37 ± 0.64	5.13 ± 2.46 ***

Data are presented as mean ± SD: standard deviation. BMI: body mass index; WHR: waist-hip-ratio; HOMA: Homeostasis Model Assessment; CRP-hs: C-reactive protein high sensitivity; HDL cholesterol: high density lipoprotein cholesterol; LDL cholesterol: low density lipoprotein cholesterol. Statistical analysis was performed with Mann-Whitney *U*-test. * *p* < 0.05; ** *p* < 0.001; *** *p* < 0.0001.

**Table 2:** Dietary intake of macronutrients and selected vitamins in obese and non-obese (control) women.

Variable	Controls (*n* = 26)		Obese (*n* = 88)	
	Mean ± SD	Median (IQR)	Mean ± SD	Median (IQR)
Total Energy (kcal/day)	1555 ± 362	1531 (1371;1676)	1922 ± 487 **	1929 (1599;2226)
Protein (% Energy)	17 ± 4	17 (14;18)	16 ± 4	16 (14;19)
Carbohydrate (% Energy)	54 ± 7	53 (50;57)	47 ± 9 **	48 (45;52)
Total Fat (% Energy)	32 ± 5	33 (31;35)	36 ± 7 *	37 (34;40)
Saturated Fat (% Energy)	11 ± 5	11 (9;12)	13 ± 3 **	13 (12;15)
Monounsaturated Fat (% Energy)	15 ± 4	16 (13;17)	14 ± 4	14 (12;17)
Polyunsaturated Fat (% Energy)	6 ± 2	6 (5;7)	5 ± 1 *	5 (4;6)
Retinol (μg/day)	398 ± 294	330 (162;509)	393 ± 260	336 (216;468)
β-carotene (μg/day)	3715 ± 2090	3566 (1891;5863)	3428 ± 1719	3146 (2041;4654)
Vitamin E (mg/day)	12 ± 6	12 (8;18)	10 ± 5 *	9 (6;13)
Vitamin C (mg/day)	120 ± 52	129 (87;157)	98 ± 57	82 (57;139)

Data presented as mean ± SD, median and IQR: interquartile range. Statistical analysis was performed with Mann-Whitney *U*-test. * *p* < 0.05; ** *p* < 0.001.

**Table 3:** Spearman correlations between DNA damage (% DNA in tail), biochemical and anthropometric parameters, and dietary intakes.

Variable	All Subjects (*n* = 114)	Controls (*n* = 26)	Obese (*n* = 88)
	*R*	*R*	*R*
Age (years)	0.018	0.420 *	0.091
Body weight (kg)	0.511 *	0.635 *	0.251 *
BMI (kg/m^2^)	0.596 *	0.551 *	0.192
WHR	0.169	0.500 *	−0.032
Fat mass (%)	0.162 *	0.598 *	0.251 *
Fat mass (kg)	0.538 *	0.670 *	0.256
Total Cholesterol (mg/dL)	0.319 *	0.297	0.183
HDL cholesterol (mg/dL)	−0.035	−0.003	0.026
LDL cholesterol (mg/dL)	0.302 *	0.248	0.155
Triglycerides (mg/dL)	0.275 *	0.493 *	0.202
Glucose (mg/dL)	0.371 *	−0.057	0.358 *
Insulin (mU/mL)	0.128	−0.515	0.045
HOMA	0.154	−0.491	0.090
Apolipoprotein AI (mg/dL)	−0.068	−0.173	0.035
CRP-hs (mg/L)	0.457 *	0.242	0.301 *
Total Energy (kcal/day)	0.446 *	0.188	0.316 *
Carbohydrate (% Energy)	−0.351 *	−0.342	−0.099
Total Fat (% Energy)	0.411 *	0.231	0.269 *
Saturated Fat (% Energy)	0.480 *	0.249	0.335 *
Monounsaturated Fat (% Energy)	−0.001	0.104	0.081
Polyunsaturated Fat (% Energy)	−0.142	−0.081	0.039
Retinol (μg/day)	−0.182	−0.492 *	−0.257 *
β-carotene (μg/day)	−0.148	−0.325	−0.193
Vitamin E (mg/day)	−0.316 *	−0.228	−0.264 *
Vitamin C (mg/day)	−0.305 *	−0.069	−0.324 *

BMI: body mass index; WHR: waist-hip-ratio; HOMA: Homeostasis Model Assessment; CRP-hs: C-reactive protein high sensitivity; * *p* < 0.05.

**Table 4:** Impact of BMI, total energy (kcal/day), SFA intake (% Energy), and vitamins C and E (mg/day) on DNA damage assessed by hierarchical linear regression analysis.

Model	B (SE)	*P*	*β*	*R*	*R* ^2^	*F*	*P*
Model 1				0.489	0.239	17.422	0.000
Age (years)	0.025 (0.035)	0.475	0.060				
BMI (kg/m^2^)	0.323 (0.055)	0.000	0.491				
Model 2				0.542	0.294	15.262	0.000
Age (years)	0.024 (0.033)	0.465	0.059				
BMI (kg/m^2^)	0.239 (0.060)	0.000	0.364				
Total Energy (kcal/day)	0.001 (0.000)	0.004	0.267				
Model 3				0.551	0.303	11.857	0.000
Age (years)	0.023 (0.033)	0.486	0.056				
BMI (kg/m^2^)	0.223 (0.062)	0.000	0.339				
Total Energy (kcal/day)	0.001 (0.000)	0.022	0.226				
Saturated Fat (% Energy)	0.079 (0.066)	0.231	0.112				
Model 4				0.571	0.326	13.199	0.000
Age (years)	0.031 (0.033)	0.354	0.074				
BMI (kg/m^2^)	0.212 (0.060)	0.001	0.323				
Total Energy (kcal/day)	0.001 (0.000)	0.007	0.249				
Vitamin E (mg/day)	−0.092 (0.040)	0.024	−0.188				
Model 5				0.587	0.345	14.354	0.000
Age (years)	0.040 (0.033)	0.224	0.097				
BMI (kg/m^2^)	0.213 (0.059)	0.000	0.323				
Total Energy (kcal/day)	0.001 (0.000)	0.009	0.235				
Vitamin C (mg/day)	−0.010 (0004)	0.004	−0.238				
Model 6				0.596	0.355	11.913	0.000
Age (years)	0.041 (0.033)	0.212	0.099				
BMI (kg/m^2^)	0.201 (0.059)	0.001	0.306				
Total Energy (kcal/day)	0.001 (0.000)	0.011	0.230				
Vitamin E (mg/day)	−0.056 (0.043)	0.189	−0.115				
Vitamin C (mg/day)	−0.008 (0.004)	0.029	−0.194				

B (SE) indicates unstandardized coefficients (standard error); *β*: standardized coefficients. Values were obtained by using multiple linear models.

## Abbreviations

CRP	C-reactive protein
WHR	waist-hip-ratio
ROS	reactive oxygen species
SFAs	saturated fatty acids
NF-kB	Nuclear factor kappa B
8-oxodG	8-Oxo-2′-deoxyguanosine
8-oxoGua	8-Oxoguanine
